# Mapping travel behavior changes during the COVID-19 lock-down: a socioeconomic analysis in Greece

**DOI:** 10.1186/s12544-021-00481-7

**Published:** 2021-03-17

**Authors:** Ioannis Politis, Georgios Georgiadis, Anastasia Nikolaidou, Aristomenis Kopsacheilis, Ioannis Fyrogenis, Alexandros Sdoukopoulos, Eleni Verani, Efthymis Papadopoulos

**Affiliations:** Transport Engineering Laboratory, Department of Civil Engineering, Faculty of Engineering, Aristotle University of Thessaloniki, Thessaloniki, Greece

**Keywords:** Coronavirus pandemic, Lockdown, Mobility patterns, Greece, Socioeconomic characteristics

## Abstract

**Background:**

COVID-19 pandemic is a challenge that the world had never encountered in the last 100 years. In order to mitigate its negative effects, governments worldwide took action by prohibiting at first certain activities and in some cases by a countrywide lockdown. Greece was among the countries that were struck by the pandemic. Governmental authorities took action in limiting the spread of the pandemic through a series of countermeasures, which built up to a countrywide lockdown that lasted 42 days.

**Methodology:**

This research aims at identifying the effect of certain socioeconomic factors on the travel behaviour of Greek citizens and at investigating whether any social groups were comparatively less privileged or suffered more from the lockdown. To this end, a dynamic online questionnaire survey on mobility characteristics was designed and distributed to Greek citizens during the lockdown period, which resulted in 1,259 valid responses. Collected data were analysed through descriptive and inferential statistical tests, in order to identify mobility patterns and correlations with certain socioeconomic characteristics. Additionally, a Generalised Linear Model (GLM) was developed in order to examine the potential influence of socioeconomic characteristics to trip frequency before and during the lockdown period.

**Results:**

Outcomes indicate a decisive decrease in trip frequencies due to the lockdown. Furthermore, the model’s results indicate significant correlations between gender, income and trip frequencies during the lockdown, something that is not evident in the pre-pandemic era.

## Introduction

In March 2020, the World Health Organization declared COVID-19 a pandemic along with the indication of Europe as the epicenter of the pandemic at that time [25]. Due to the lack of effective treatments or vaccines, European countries began taking social distancing measures to control the spread of the disease. In most European countries, social distancing measures started as advisory ones, but soon turned into countrywide lockdowns. The closure of educational institutions, shops, restaurants as well as the cancellations of mass gatherings and the encouragement of home-based teleworking significantly reduced travel demand and affected travel habits and preferences, since health-related concerns also emerged [50]..

Social distancing, also called physical distancing, is a set of non-pharmaceutical interventions or measures aimed at preventing the spread of a contagious disease by maintaining a physical distance between people and reducing the number of times people come into close contact with each other. It usually involves keeping a certain distance from others (the set distance may vary from time to time and from country to country) and avoiding gathering together in large groups [50]. While, social distancing aims to break the transmission of the pandemic by restricting or closing all public places such as cafes and malls, nation or countrywide lockdowns are a stricter strategy that involves the complete interruption of all passenger trips except for essential services.

The impact of different COVID-19 confinement policies on how mobility characteristics have changed after the spread of COVID-19 has not been studied yet to a large extent. Based on preliminary analysis and results from different countries it can be argued that the implementation of social distancing measures had a significant effect on personal mobility [6]. Societies were not sufficiently prepared to deal with a pandemic and implemented movement restriction measures that were often harsh, unbalanced and unheated creating confusion, uncertainty and annoyance to communities [18]. While lockdowns may vary among countries, both in terms of measures implemented and in how strictly these measures were enforced, a central tenet has been the restriction or suspension of transport and mobility services. In the most extreme cases, such as in Egypt and India, all transportation has been banned—including private vehicles—and strict, since universal police enforcement has been in place [44]. Generally, travel demand has declined, and many countries have seen dramatic reductions in car traffic and in public transport ridership (often resulting in less frequent services) [36]. Vehicle traffic volumes have been greatly reduced worldwide. In the United States, vehicle volumes fell by 41% from pre-pandemic levels [43]. People avoided public transport as it was considered fertile ground for viruses and places where it might be difficult to avoid contact with other passengers [47]. In almost every country, public transport ridership has decreased in response to stay-at-home orders and fear of the virus. Passenger traffic in some cities’ ridership has been reduced by more than 90% [19]. Those who had no choice but to use public transport tried to avoid crowded buses and trains by travelling during off-peak hours. Bicycle use soared at the onset of the virus as people sought a safe, reliable mobility option, and it has remained a popular choice for travel, especially for short or recreation trips. People preferred home-delivery of goods purchased online (e.g., food, clothes), resulting in less shopping trips. Global freight demand has generally declined, but local deliveries below 100 miles have been increased by 100% as residents have sheltered-in-place and retail has closed [45]. On the other hand, people with access to a car, began to drive more, as the car “protected” them from other travelers. On the positive side, reduced demand for (motorized) transport resulted in fewer road accidents (and related injuries and fatalities), and safer walking and cycling conditions [37]. Several cities have already reported significant reductions in traffic accidents, although the share of speeding cars is often reported to increase [41]. In addition, reduced traffic might lower air pollution, resulting in reduced chances of respiratory diseases, asthma, lung damage and high blood pressure [51], and possibly slowing down global warming. Pedestrian volumes have been decreased in the densest corridors, but new destinations have led to the need for more open space to ensure compliance with social distancing measures.

However, the decline in human mobility during lockdown occurred at different scales based on socioeconomic characteristics, such as age, gender, income, education, etc., of different population groups. Different rates of reduced mobility across different socioeconomic characteristics and levels may affect the effectiveness of the standard epidemiological containment policy based on lockdown and social distancing measures. Associations of mobility reduction with demographic and socioeconomic indicators would help identify population groups for whom the consequences of the COVID-19 measures are greater than for others. The effects of COVID-19 are many and pervasive, from social, economic, to environmental. Certain populations experience differential exposure and extensive corresponding effects. For example, elderly people with chronic illnesses such as heart disease, diabetes, and lung disease, are more likely to be affected by the virus. People with disabilities face differential access, risk, and consequences. The pandemic crisis will also widen the gap between people in society who have opportunities and those who do not. It is very likely that those already in a more vulnerable position will be more affected by the COVID-19 measures, due to financial uncertainty and less access to different mobility options [28]. The International Labour Organisation estimates that COVID-19 could cause the equivalent of 305 million full-time job losses worldwide. Quarantined low-income communities who cannot work will not be able to afford basic necessities - food, water, and non-toxic sanitation supplies. They may also need assistance with energy bills /or rent. Furthermore, women are particularly vulnerable in a pandemic situation, due to their general disadvantages in transportation and a high share in society’s care work [23].

Based on the literature review, it can be witnessed that the impact of the coronavirus on mobility rates seems to be greater among women, especially women with a lower level of education [26]. The percentage of women who stayed at home or were temporarily unemployed due to the COVID-19 crisis is higher compared to men. Globally, women are likely to experience a significant burden given their multiple care responsibilities due to school closures and confinement measures adopted, possibly resulting in reduced working time and permanent exit from the labor market. Women are overrepresented in professions that can/may not be performed temporarily such as beauticians, hairdressers, cleaners or administrative employees. It can also be expected that women will have fewer alternatives at their disposal, compared to men. In particular, it was found that first-generation, less educated women with a non-Western background and single mothers are more likely to experience mobility problems, because they have few alternatives to urban public transport [46]. Less educated people generally had fewer opportunities to work during this period and therefore travel much less. As a result, there is not only greater uncertainty for less educated women during the COVID-19 crisis, but also a risk of financial hardship and social isolation [32].

On the other hand, the impact of lockdown on the reduction of outgoing mobility can be strongly correlated with the population fraction of the most active age group. Countries, such as China, South Korea, Italy, and Iran, with a high percentage of the population in the age range of 24–59 years old were also the ones where lockdown had the greatest impact on mobility [38]. Besides the displacements to go to work, the specific population group is also highly mobile for leisure activities, which were completely banned by restrictions. In addition, there has been a sharp decline in mobility rates for older people, who are at higher risk of developing severe forms of COVID-19 if infected. The elderly almost stopped making longer trips, avoided leisure activities and family trips, as recommended by the authorities [2].

Finally, the decline in human mobility during lockdown occurred at different rates for high versus low-income groups in most countries, as the mobility rates in the higher-income groups were higher than in the lower-income groups. This phenomenon is known as the mobility gap [12]. In particular, low-income employees to a greater extent can no longer engage in working activities and they do not need to leave home on a daily basis [21, 30]. Research in the United States has shown that workers in low-income deciles are less able to work from home than those in higher deciles, and are disproportionately affected by extensive lockdowns [40]. In Italy, the decline in connectivity and mobility is higher for low-income municipalities, while high-income municipalities experience less drastic changes. The mobility gap seems to be a widespread, but not universal, phenomenon that occurs mainly in more densely populated urban centers [38].

Based on the above, health and mobility authorities need to understand whether (and in what extend) social distancing policies and lockdown measures have the desired impact on peoples’ mobility, since reduced social interactions achieve lower transmission and mortality rates. In addition, these policy measures have high social and economic costs, so they cannot last indefinitely and there is a need for a continuous evaluation of what interventions are necessary to maintain control of social distancing. Understanding what works, when and how regarding the specific characteristics of the various population groups, is also crucial to answering the question when and how the restraint measures can be relaxed.

In this context, the present study attempts to shed some light on: (a) how lockdown measures affected key personal mobility attributes as well as (b) the impact of socioeconomic characteristics on personal mobility changes during the first wave of the COVID-19 pandemic in Greece. Greece is an interesting case, as it reacted relatively early and decisive. The measures implemented in Greece are among the most proactive and strictest in Europe and have been recognized internationally for having slowed the spread of the disease and having kept the number of deaths among the lowest in Europe. COVID-19 pandemic and the subsequent potential economic consequences, occurs at a time when Greece is still struggling to recover from the financial crisis of 2007–08, during which the Greek economy suffered the longest recession of any advanced capitalist economy until today, overcoming the US Great Depression [7]. As a result, the Greek political system has been upended, social exclusion has risen, and Greece’s unemployment rate remains the highest in the euro zone. The social and economic situation is difficult for the average Greek citizen, apart from the recent healthcare threat. Βased on the special characteristics of Greek society as well as the fact that many cities and regions of Greece are located in those areas of Europe with the lowest birth rate, indicating an increasing part of ageing population [14], it is of particular interest the study of how Greek people responded to social distancing measures. Therefore, the research questions that this study seeks to answer are as follows:

1. What was the impact of COVID-19 lockdown measures on general mobility characteristics, such as trip frequency and transport mode choice, in the case of Greece?
2. How did main socioeconomic characteristics, such as gender, age, income, educational level etc., influence mobility behavior and perceptions during the COVID-19 lockdown period in Greece?

To meet the above research objectives, this paper presents and analyzes questionnaire survey results regarding how lockdown measures affected the mobility profile of different population groups in Greece. The potential association of mobility reduction with demographic and socioeconomic characteristics could help policymakers tailor their agendas in favour of population groups, which were mostly affected by the consequences of the COVID-19 pandemic. The remainder of this paper is structured as follows. On the next section a brief summary of the spread of the pandemic in Greece is presented, as well as the timeline of the social distancing measures that the government introduced. In Section 3, the design of the questionnaire survey, sampling details, as well as the data analysis methods, are explained. The results of the statistical analysis performed in the present research are discussed in Section 4. Finally, the main conclusions of this research are summarized in Section 5.

## The case of Greece in the first wave of the COVID-19 pandemic

In Greece, the first laboratory – confirmed coronavirus case and the first coronavirus death, were reported on 26 February 2020 and 12 March respectively. As of July 5th, the total number of confirmed coronavirus cases and the total number of deaths from the COVID-19 disease in Greece were 3519 and 192 respectively. Also, of the total of 3519 cases, 816 (23.2%) were related to travel from abroad, 1933 (54.9%) were related to an already confirmed case and the rest are under investigation. The average age of confirmed cases was 47 years old and the average age of fatalities was 76 years old, while 55% of the confirmed cases were men. The peak of daily active cases was reported on 21 April 2020 with 156 active cases. In addition, the peak of deaths per day occurred on 4 April 2020 with 9 new deaths [52].

Until the 5th of July, Greece, with only 328 total confirmed cases per million inhabitants and 18 totals deaths per million inhabitants, recorded one of the lowest counts both in the EU and globally. More specifically, at global level Greece ranked 96th (out of 218 countries with confirmed COVID-19 cases) and 21st in relation to the other 27 countries of the European Union [16].

Greek government undertook measures to control COVID-19 pandemic while the number of cases was still low. The measures implemented evolved gradually from soft (encouragement of physical distancing, restrictions on public gatherings, movements restrictions in specific areas) to drastic (lockdown measures in national level). As the COVID-19 crisis was escalating, Greek government decided to take more strict measures. The countrywide level lockdown measures were adopted on March 23rd. Citizens could move only for specific purposes: a) commuting, b) trip to a pharmacy or healthcare services, c) trip to an essential goods stores, d) trip to a bank, e) trip to a ceremony (wedding, funeral), f) outdoors physical exercise or pet walking. These designated movement had to be authorized by an SMS message or a handwritten document, in which citizens declared the trip purpose (one of the above), their name and the address of their permanent residence. Citizens were required to carry their ID or passport with them, as well as the corresponding certification (SMS or handwritten document) explaining the purpose for their trip. Police patrols were continuously monitoring the proper implementation of movement restriction rules and had the authority to impose fines for those who did not comply with the lockdown measures. Also, all the hotels and recreational facilities (e.g. cinemas, thematic parks etc.) were closed. In addition, the maximum number of passengers in vehicles was set to 3 (including the driver). At the same time remote work and work from home was encouraged. Intercity and international passenger trips were prohibited while public transport services were limited.

**Fig. 1:**
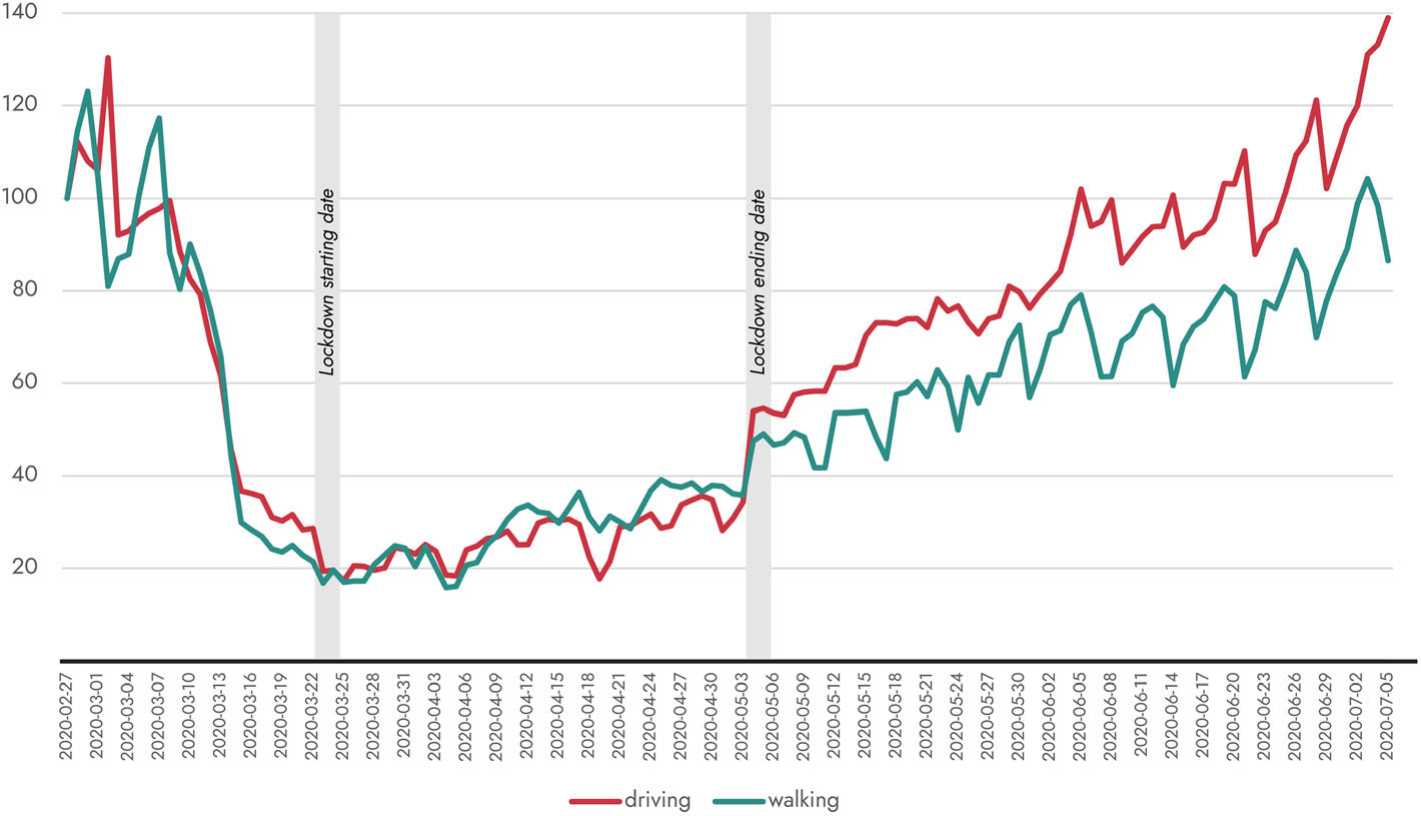
Mobility Trends Reports for Greece from 27/02/2020–05/07/2020

Table 1 presents the timeline of the social distancing and lockdown measures taken in national level by the Greek government in relation to total confirmed cases and deaths reported for Greece. In Fig. 1, mobility trends for driving and walking during the countrywide lockdown period (March 23 – May 4) as well as during the relaxation of the imposed restrictions are presented [3]. During this reference period (February 27 – July 5), mobility patterns of Greek citizens varied widely in line with the traffic restriction measures before, during and after the lockdown event. Driving shows the highest decrease during the first weeks of the countrywide lockdown period while after the relaxation of measures it gradually increases and returns to the levels of a typical day. On the other hand, walking also presents the highest decrease in the same period, similar to the driving one. However, a higher share of trips made on foot is observed at the end of February when the first social distancing measures took place. During the lockdown period walking rates are very low manifesting a general reluctance of citizens to move regardless of transport mode.

**Table 1:** Timeline of measures adopted by the Greek government to control the first wave of COVID-19 pandemic

Type of measure	Date	Total confirmed cases	Total deaths	Measures taken
Social distancing measures	Feb-27	3	0	Cancellation of carnival events countrywide
	Mar-10	89	0	Close-down of educational institutions
	Mar-12	117	1	Close-down of playgrounds, theatres, cinemas, courtrooms, gyms
	Mar-13	190	1	Close-down of shopping centres, cafes, restaurants, bars, museums and archaeological sites, hairdressing and other beauty treatment, sports facilities
	Mar-14	228	3	Close-down of organized beaches and ski resorts
	Mar-16	352	4	Close-down of places of worship
	Mar-18	418	5	Close-down of retail sector (except for food stores, pharmacies etc.)
	Mar-19	464	6	Prohibition of gatherings up to 10 people in public and private spaces
Lockdown	Mar-23	695	17	Imposition of countrywide level lockdown measures. Close-down of hotels. Introduction of movement authorization via SMS.
Relaxation period	May-04	2627	146	Partial relaxation of traffic restrictions and reopening of businesses (10% of businesses that were put in suspension). Suspension of SMS movement restriction scheme.
	May-11	2720	151	Reopening of businesses (25% of businesses that were put in suspension), partial reopening of schools (only for the last grade of high school)
	May-17	2828	163	Release of all traffic restrictions. Partial reopening of schools (secondary education). Resumption of religious events, malls, museums and archaeological sites
	May-25	2875	172	Reopening of outdoor catering businesses
	Jun-01	2917	179	Reopening of all educational facilities (except for universities). Reopening of indoor catering businesses. Relaunch of tourism businesses
	Jun-15	3132	184	Reopening of sports facilities. Re-establishment of international flights
	Jul-01	3,43	192	Opening of borders for tourists except for tourists from countries with a high number of confirmed cases

## Methodology

### Survey characteristics

In order to meet our research objectives, a questionnaire survey was structured and distributed through nationwide and local news outlets, between 6 and 19 of April of 2020. This certain survey distribution time-window, which coincides with the 3rd and 4th week of the lockdown period, was chosen, in order to highlight the trip preferences of citizens after the adjustment period to the new mobility restraints. The survey was designed with the exploitation of an online survey service, since the collection of responses through face to face personalised interviews, was not possible because of the lockdown prohibitions. In total, 1259 of the collected questionnaires were considered valid and processed further for our analysis. The representativeness of the survey’s sample was assessed based on the control variables of age, gender and residence location. Eq. 1 presents the formula for the margin of error (MOE) which was calculated at 2.76., if we take into account a confidence level of 95% and total population size of 10,724,599 citizens, i.e. the current estimated population of Greece [15].

1 $$ MOE={z}_{\gamma}\times \sqrt{\frac{\sigma^2}{n}} $$

Where:

*n*: is the sample size of the survey, equal to 1259 cases.

*z*_*γ*_: is the quantile (critical value) for confidence level of 95%, equal to 1.96.

*σ*: is the standard deviation percentage (distribution) of the given answers, assuming a pessimistic value of 50%.

The composition of the sample, as well as its representativeness against the control variables, are summarized in Table 2. Regarding age, the sample is distributed equally on most of the age clusters, except the 60–69 cluster, where there is a difference of nearly 9%. The difference could be justified by the fact that the survey was distributed through an online service, which requires a level of familiarity with the Internet, something that is not common in older respondents (over 55 years old) who tend to use the Internet less [13]. The composition regarding the urban centre where respondents were living during the lockdown period, shows certain deviations. The low sample percentage in cities with population between 10,000 and 100,000 citizens, could be justified by the great number of municipalities that belong to this category (227 out of the total of 315 municipalities of Greece), making it extremely difficult to send the questionnaire to all potential local media in order to attract as many respondents as possible. On the other hand, higher sample percentages in large urban centres with population over 100,000 citizens, could be justified, by the wider access to digital information, through web-based news nationwide outlets, where the questionnaire was mainly distributed. Also, in large urban centers live mainly younger people and therefore more familiar with technology and the use of internet.

**Table 2:** Sample composition and representativeness

Variable	Levels	Sample (%)	Population (%)
Gender	Male	48.02	49.03
	Female	51.83	50.97
Age (years)	15–19	2.74	1.00
	20–29	17.35	19.00
	30–39	27.44	23.00
	40–49	25.59	22.00
	50–59	19.61	19.00
	60–69	7.26	16.00
Urban Population (citizens)	< 2000	6.43	0.23
	2000-10,000	8.81	2.84
	10,000-50,000	19.29	38.95
	50,000–100,000	22.86	30.76
	> 100,000	42.62	27.21

### Survey design

**Fig. 2:**
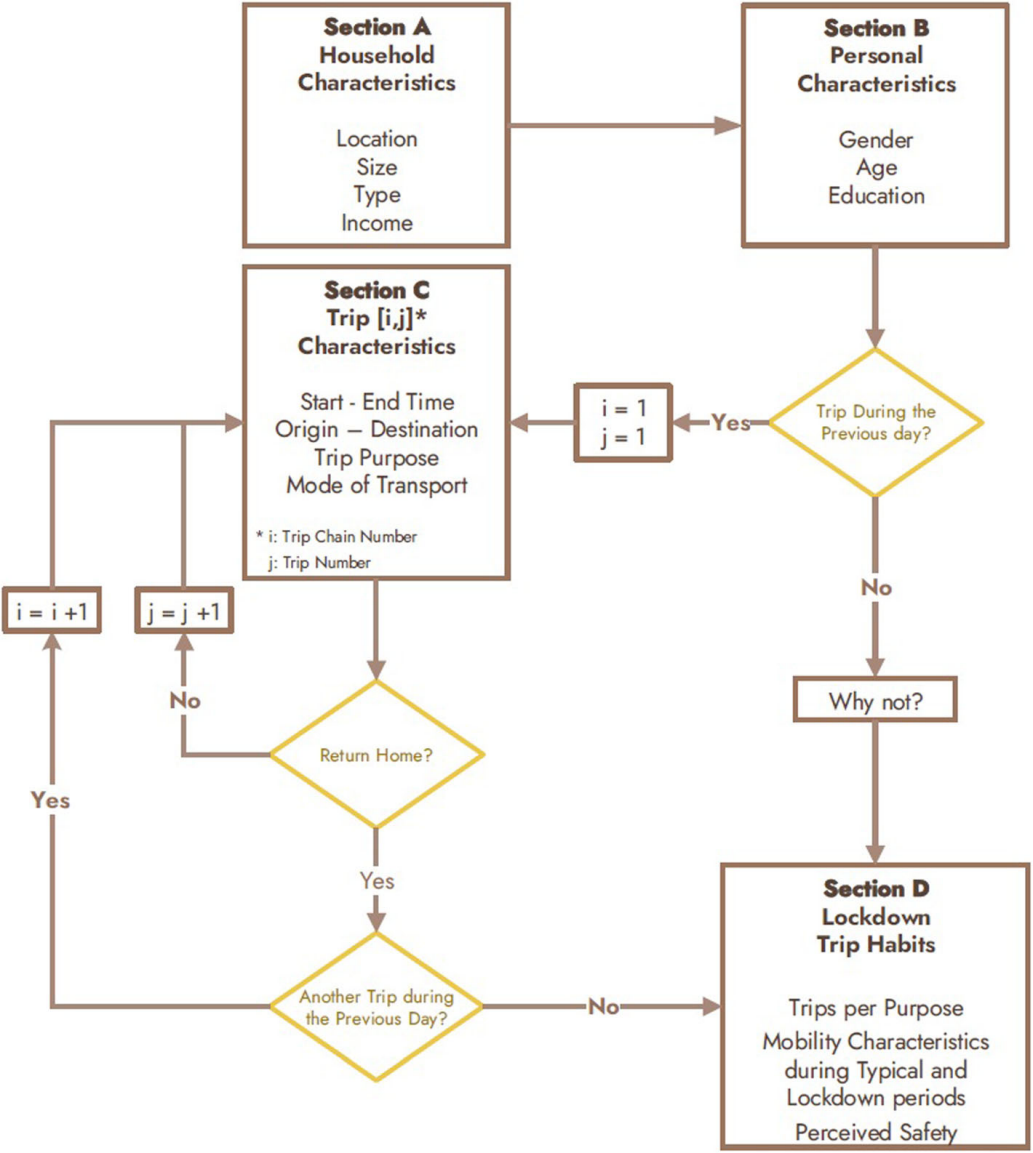
Flow chart of survey’s design

The questionnaire was divided in 4 sections. Their sequence and the logic by which they were presented to the respondents is shown in the flowchart of Fig. 2. At Sections A and B, respondents were asked to answer specific descriptive questions regarding their household and personal socioeconomic characteristics. Following this, respondents were asked whether they made a trip during the previous day, and if not, the reasons they did not. The respondents who did make at least one trip were asked to describe certain characteristics of the trip in Section C, such as the start and end time of the trip, the trip purpose, the mode of transport and its duration. Afterwards they were asked if they continued to another destination or if they returned home and made any subsequent trips during the same day. By this way, it was possible to map the specific trip chains the respondents made and collect the trip characteristics for each Trip [i,j], where i is the number of the trip chain and j is the number of the trip within the trip chain. Finally, in Section D respondents were asked to describe certain aspects of their daily trips (e.g. transport mode preference, number of trips per trip purpose, perceived level of safety etc.) under typical conditions and during the lockdown period. Table 5 and Table 6 of Appendix, present the descriptive statistics for the scale and nominal/ordinal variables respectively, which were quantified by this questionnaire survey and used further in the analysis of the study.

### Analysis setting

In order to examine our first research question, we considered the “total number of trips” (Table 5 in Appendix) and the “transport mode” (Table 6 in Appendix), per trip purpose, as main travel behavioral variables and examined their relationship between lockdown period and typical circumstances (before lockdown) in Greece. In our analysis we assessed correlations at 3 significance levels (95%, 99% and 99.9%), with the use of appropriate inferential statistical tests. Table 3 presents the framework of this statistical analysis as well as the hypothesis setting regarding each statistical test (#1 and #2 of Table 3). The selection of the appropriate statistical tests was based on the type of variables examined each time [49].

**Table 3:** Initial hypotheses and test statistics used for investigating the relationships between mobility variables and socioeconomic characteristics

#	Dependent variables	Independent variables	Hypothesized relationship	Statistical Test
1	Total number of trips (per trip purpose)	Reference period (typical:0; lockdown:1)	Reduced number of trips during pandemic for all trip purposes	Independent-samples t-test
2	Transport mode (commuting and shopping)	Reference period (typical:0; lockdown:1)	Reduction of public transport modal shares during pandemic	Chi-square test
3	Trip purpose during pandemic	Age Groups	Older aged people make less trips for commuting and shopping.	Spearman’s rho correlation test
4	Transport mode (commuting and shopping) during pandemic	Age Groups	Ambiguous	Kruskal – Wallis correlation test
5	Transport mode (commuting and shopping) during pandemic	Gender	Ambiguous	Chi-square test
6	Perception of Safety	Gender	Lower safety perception among women	Chi-square test
7	Perception of Security	Gender	Lower security perception among women	Chi-square test

For our second research question we utilized two analysis methods:

- Firstly, we employed inferential statistical analysis to investigate whether certain mobility behavioral variables, i.e. “transport mode” (per trip purpose) and “trip purpose” during lockdown (Table 6 in Appendix), as well as travel perception variables, i.e. “perception of safety” and “perception of security” (Table 6 in Appendix), were significantly differentiated across age group and gender characteristics of the respondents in our sample. Table 3 presents the five statistical tests and initial hypotheses that were considered to examine the above relationships (#3 to #7 of Table 3).
- Secondly, we developed suitable regression models, derived from the wider class of Generalized Linear Models (GLM) [31], to model the effect of socioeconomic attributes in the trip frequencies before and during the lockdown event (pre-pandemic and pandemic period). Therefore, we estimated two (2) GLMs, examining the Total Number of Trips (dependent variable) for all trip purposes, against age, gender, income and educational level (independent variables) before and during the lockdown period. The most appropriate GLM model was selected based on the distribution of the chosen dependent variables and the type of the collected data. The normality of the two dependent variables was tested through the Shapiro-Wilk test. Results showed that both variables were not normally distributed (*p* = .000 < 0.05). Additionally, both variables did not fulfil the criteria of Poisson distribution, since the mean value equal to variance criterion was violated in both cases (Table 5 in Appendix). Considering the above, linear and Poisson regression models were deemed as unsuitable. Furthermore, if we take into account that both dependent variables are characterized by non – negative, over – dispersed count data, a negative binomial regression model was finally considered as the most appropriate one [49].

All inferential statistical analyses and GLMs calculations were done with the use of IBM SPSS software [22].

Το develop the hypothesis setting for our research (Table 3), we considered the existing literature regarding the effect of the pandemic on mobility and the influence of certain socioeconomic characteristics on the mobility patterns during that period. More specifically:

- Research has shown that the total number of trips has been reduced significantly since the start of the pandemic, as a result of the imposed social distancing measures by governments [6, 11]. Especially for public transport, where crowding is often observed, figures indicate a reduction in ridership, with passengers preferring more private transport modes, such as private cars, or active modes, i.e. cycling or walking [5, 11, 18, 34]. For these reasons, we expect that both total number of trips and public transport modal shares would be reduced during the pandemic period (#1 and #2 of Table 3).
- Past research results on the relationship between age and trip purpose, indicate that younger users (18–35 years old) performed more trips across all purposes, while older users (over 55 years old) tend to make less trips than younger users for commuting and for shopping [4]. In this respect we have set accordingly our initial hypothesis (#3 of Table 3).
- Regarding mode choice and socioeconomic profiles, although literature suggests that the elderly would avoid public transport amid the existence of an outbreak [10], there is not enough evidence to explain the mode choice of other age groups. Concerning the influence of gender on transport mode choice during the pandemic, males are more likely to choose private car than females, although evidence regarding other modes is not robust [1] yet. Therefore, we considered the hypothesized relationships among these variables as ambiguous (#4 and #5 of Table 3).
- Previous literature findings underlined that females are more hesitant in traveling during a pandemic compared to males, due to their increased concern regarding the spread of the disease [9]. Females were also more reluctant in traveling during COVID-19 crisis according to reports, due to the lower number of people on the streets contributing to potentially increased risk of exposure to criminal activity and sexual violence [48]. As a result, we expect that women would express comparatively lower safety and security perception levels than men (#6 and #7 of Table 3).

## Results and discussion

### COVID-19 lockdown effect on general mobility characteristics

**Fig. 3:**
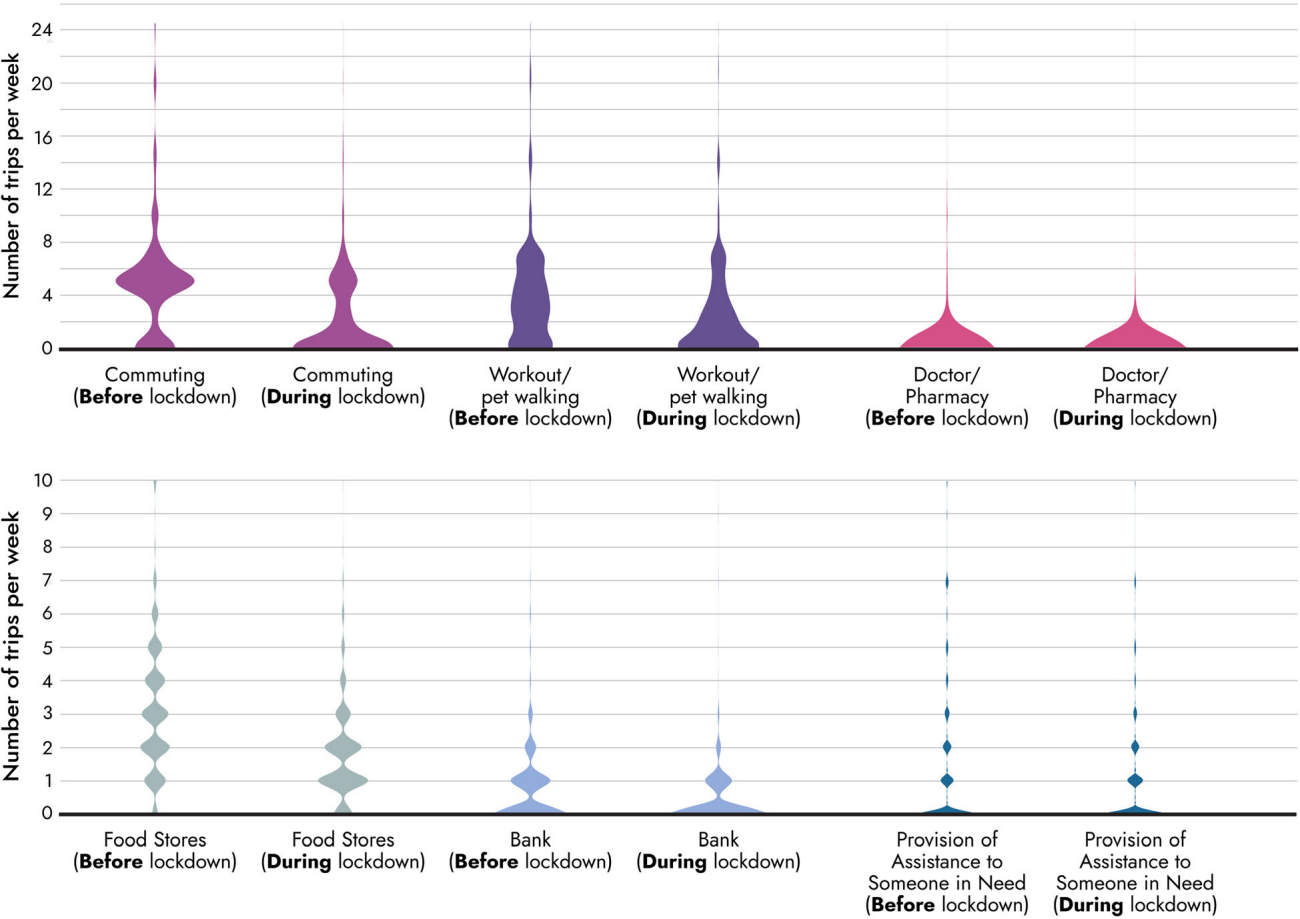
Trip frequencies before and during the lockdown period per trip purpose

In general, a reduction in the number of weekly passenger trips before and during the lockdown period is observed across all the main trip purposes (commuting, workout, shopping) by 51% (Table 5 in Appendix). An independent-samples t-test was conducted to compare the total number of weekly trips before and during the lockdown period. There was a significant difference in the scores before (Mean = 15.878, SD = 13.269) and during the lockdown period (Mean = 7.678, SD = 0.217) conditions; t (25) = − 18.968, *p* = 0.000. Independent samples t-test were also conducted for the total number of trips before and during the lockdown for all trip purposes and there were significant differences in the trips made for all trip purposes. This outcome is in line with our initial hypothesis on total number of trips (Table 3). In Fig. 3, the trip frequencies before and after the imposition of the lockdown period for 6 trip purposes, are presented.

The largest decrease, approximately 62%, was observed for commuting purposes. This is possibly due to the increased rate of teleworking or the significant number of enterprises that remained closed during lockdown. The percentage of Greek employees working from home was 5% in 2015 while during COVID-19 crisis 26% of employees in Greece started working at home and nearly 95% of Greek companies introduced teleworking schemes [24]. On the other hand, 14.6% of Greek businesses have been suspended under a state mandate. In total, these enterprises employed 1,063,098 employees, which means that 25.4% of Greek employees were temporarily unemployed during the lockdown period [20]. A significant proportion of shopping and outdoor walking trips has also decreased, indicating a high sense of insecurity among citizens and at the same time a sense of compliance with the general restrictions.

**Fig. 4:**
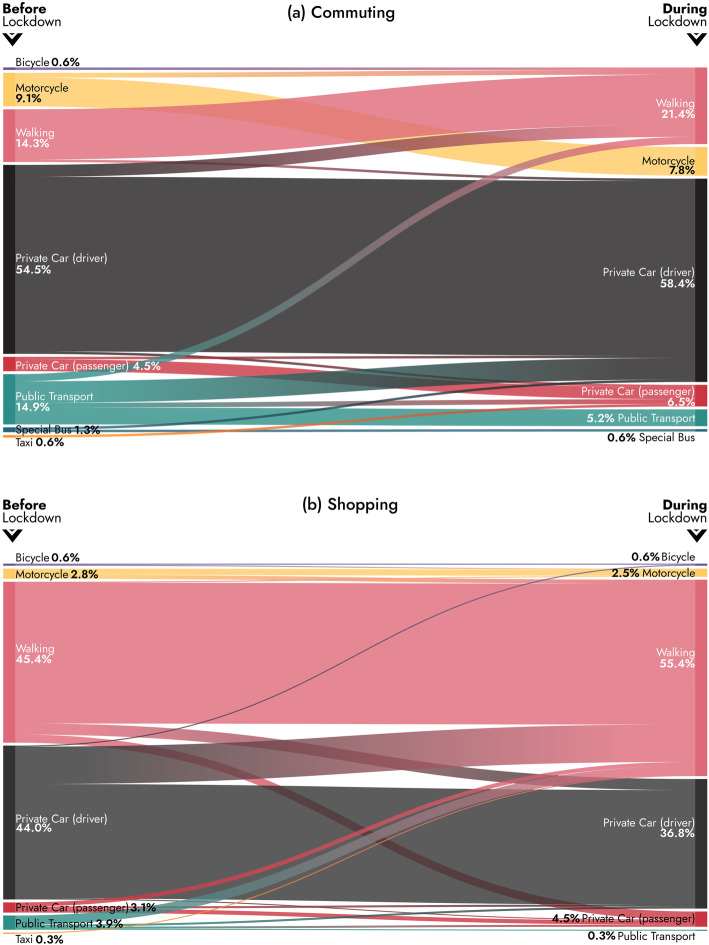
Flows between transport mode use before and during the imposition of lockdown measures for commuting and shopping

A chi-square test of independence was performed to examine the relationship between the transport mode used for commuting and shopping during the lockdown period and before, i.e. during a typical week. The relationship between these variables was significant, X2 (49, *N* = 533) = 1665.56, *p* = .000 for commuting and X2 (36, *N* = 1080) = 2380.96, p = .000 for shopping. Figure 4 illustrates the mode shift of users, before and after the lockdown measures, for commuting and shopping. During lockdown there was an increase in the rate of use of the private car for commuting as well as on pedestrian trips both for commuting and shopping. The use of public transport was significantly reduced. This is possibly explained by the limited capacity of buses based on health guidelines, due to the government recommendations for the greatest possible reduction in public transport journeys and also due to the fear of potential exposure to COVID-19. As a consequence, public transport users switched to more private transport modes and walking mainly for commuting purposes. This result comes in agreement with our initial hypothesis on transport mode (Table 3). A considerable share of private car users shifted to walking for shopping purposes during the lockdown. Concerning other transport modes, bicycle use remained very low even during lockdown, unlike other cities and regions where there has been a sharp increase in recreational cycling during the pandemic that has led local authorities to increase the overall length of existing cycle paths. In fact, more than 150 cities, such as Berlin, Bogota, Mexico City and New York have deployed emergency cycling infrastructure as of late April 2020, with many hundreds more planning to do so as confinement is eased [33].

### Socioeconomic influence on mobility behavior and perceptions

#### Trip frequency

Table 4 summarizes the GLM results, which associate the total number of trips per person during a typical week and during a week in the lockdown period for two discrete residential clusters, i.e. cities populated over and below 100,000 citizens, against a set of socioeconomic variables. The two clusters are defined by the population threshold of 100,000 inhabitants, which is mainly associated with different characteristics that a large urban city and a smaller semi-urban city or settlement usually have, in terms of transport supply (e.g. public transport provision) and mobility behavior. For instance, public transport usage and coverage may considerably differ between a large urban center and a smaller city in Greece thus influencing modal split and travel behavior in general. Additionally, urban areas, especially large urban centers, have been affected the hardest by COVID-19. In fact, there are four main explanations linking urban areas and coronavirus pandemic spread, emphasizing density; connectivity; crowded living conditions; and exposed occupations [29].

**Table 4:** Results of total number of weekly trips per person in the typical & lockdown periods for 2 population clusters

Variables	Typical Period				Lockdown period			
	Unstandardized Coefficients			Sig.	Unstandardized Coefficients			Sig.
	Β	exp(B)	Std. Error		Β	exp(B)	Std. Error	
**Population Cluster: Over 100,000 citizens**								
Gender:								
Male	–	–	–	–	0.265	1.303	0.0615	0.000^a^
Age Groups:								
26–40	0.170	1.186	0.0700	0.015^a^	0.422	1.525	0.1174	0.000^a^
41–64	0.288	1.334	0.0693	0.000^a^	0.541	1.718	0.1164	0.000^a^
> 65	0.193	1.213	0.1063	0.070	0.475	1.608	0.1739	0.006^a^
Monthly Average:								
1000-2000€	–	–	–	–	–	–	–	–
> 2000€	–	–	–	–	–	–	–	–
Intercept:	2.354	10.529	0.0635	0.000^a^	1.260	3.525	0.1109	0.000^a^
	Model Summary-Goodness of Fit Metrics							
	Omnibus Test		0.000		Omnibus Test		0.000	
	AIC		3340.718		AIC		2961.605	
	BIC		3362.120		BIC		2987.287	
**Population Cluster: Below 100,000 citizens**								
Gender:								
Male	–	–	–	–	0.318	1.375	0.056	0.000^a^
Age Groups:								
26–40	0.355	1.426	0.0544	0.000^a^	0.522	1.685	0.1006	0.000^a^
41–64	0.326	1.385	0.0525	0.000^a^	0.575	1.777	0.0995	0.000^a^
> 65	0.140	1.150	0.1154	0.226	0.564	1.758	0.1907	0.003^a^
Monthly Average:								
1000-2000€	–	–	–	–	− 0.154	0.814	0.0649	0.007^a^
> 2000€	–	–	–	–	− 0.205	0.857	0.0757	0.017^a^
Intercept:	2.281	9.784	0.0462	0.000^a^	1.369	3.931	0.0945	0.000^a^
	Model Summary-Goodness of Fit Metrics							
	Omnibus Test		0.000		Omnibus Test		0.000	
	AIC		4595.488		AIC		3494.029	
	BIC		4618.398		BIC		3529.376	

*Note 1: Dependent Variable: Total Number of weekly trips per person in typical and lockdown conditions*

*Note 2: Reference categories: Female for Gender, 18–25 years old for Age Group and less than 1000 euros for Income*

^a^*Significance at 1%*

Table 4 presents the four distinct GLMs formulated for the two population clusters and the two reference periods (typical, lockdown). The statistically significant variables are flagged with asterisks (*/** statistical significance at the alpha = 0.05/0.01 level). The goodness-of-fit statistics showed an acceptable fit of the proposed GLMs for both periods and population clusters. The *p*-values (Sig.) associated with the Omnibus Test appeared to be smaller than the alpha level (0.000 < 0.05), for all models, indicating that all the independent variables collectively improve the model over the intercept-only model (i.e. with no independent variables added). The Akaike’s Information Criterion (AIC) and Bayesian Information Criterion (BIC) appeared to be relatively small for all models, suggesting that they fit well the observed data.

Based on Table 4 results, men are associated with increased trip frequencies during the lockdown period when compared to women, a difference that is not observed during the typical week. In cities with over 100,000 citizens, men are 30.3% more likely to perform more trips than women, while in cities with population of less than 100,000 citizens this percentage difference is 37.5%. These results indicate that lockdown could have an uneven effect on mobility across gender and would impact women more strongly. Firstly, disease outbreaks increased women’s duties caring for elderly and ill family members, as well as for children who are out of school [35]. Beyond this, restrictive and social distancing measures implemented around the world could possibly impose a threat upon several women-dominated industries. This includes air travel, tourism, retail activities, accommodation services (e.g. hotels), and food and beverage service activities (e.g. cafés, restaurants, and catering). Many of these industries are major employers of women: on average across OECD countries, women make up roughly 47% of employment in the air transport industry, 53% in food and beverage services, and 60% in accommodation services. In the retail sector, on average, 62% of workers are women [39]. On the other hand, men were not affected at the same extent as women since they typically travel by car, while women use public transport more than men [8]. Due to COVID-19 restrictions, public transport service supply was limited, as a result of health experts’ guidelines and government recommendations, thereby reducing women’s ability to travel with the specific transport mode. During the pre-pandemic period, gender doesn’t seem to affect significant the total number of trips made for both population clusters.

The effect of respondents’ age on the total number of trips is statistically significant for both time periods and population clusters. During the pre-pandemic period, the age group of 41–64 tends to travel more compared to the other age groups in large cities, while in small-sized cities the age-group of 26–40 appears to make more trips. During the lockdown period, for both population clusters, people belonging to the age group of 41–64 are more likely to make more trips compared to the other age groups. Table 4 results show that the mobility drop, due to the COVID-19 outbreak, was considerably stronger for younger people (18–25) due to the stay-at-home orders, reduced leisure activities, schools and universities closure. Younger people stayed more at home, compared to other age groups, also due to the fact that they are less likely to be employed or because they are mainly employed in the food and beverage sector, a sector that was strongly influenced by the lockdown in Greece, as restaurants and coffee shops were closed or operated for less hours and with limited staff. Additionally, younger people are more likely to perform activities, such as shopping, from the safety of their home with the use of internet technology. People between ages of 26–64 were found to travel more during the lockdown period compared to the younger ones and this can be associated with the fact that people of this group were more likely to travel for work or shopping purposes [27]. In general, it seems that older travelers (> 65 years old) generally maintained their pre-pandemic mobility behavior patterns and did not sufficiently comply with the general instructions to reduce non-essential trips to minimum, despite their higher vulnerability to COVID-19. This could be probably explained with the low level of familiarization with technology among the elderly, which would dissuade them from exploiting internet banking or online shopping applications and consequently force them to travel in order to complete these activities. It should be also noted that the relatively high coefficients for the age of “> 65″ for both time periods and population clusters, may be affected by the few number of respondents belonging to the specific age group.

In respect to the monthly average household income of respondents, income groups were found statistically significant only for the model that represents the total number of trips made in cities with population below 100,000 citizens and only during the lockdown period. In the specific model, the reference category of “< 1000€” was the only income group that was found to be associated with more total trips compared to the other income groups. People from lower-income groups seem that they have not reduced their trips in the same degree applied to other income groups, probably because they cannot work from home as easily and also because they are less likely to shop online [42].

Educational level was also examined along with the above socioeconomic variables, but no statistically important effect was demonstrated in all four models which were developed.

#### Trip purpose

Regarding the trip purposes of respondents with regard to their age group classifications, our statistical tests showed that the people who belonged to the 30–39 age group performed more commuting trips compared to all other age groups during the lockdown period in Greece (Spearman’s rho correlation test, rs = 0.087, *p* = 0.002. *N* = 1259). This is probably explained due to the fact that younger people are not having the same level of fear or anxiety when moving outside home [17]. No other statistically important differentiations were found between trip purpose and age groups in our sample.

**Fig. 5:**
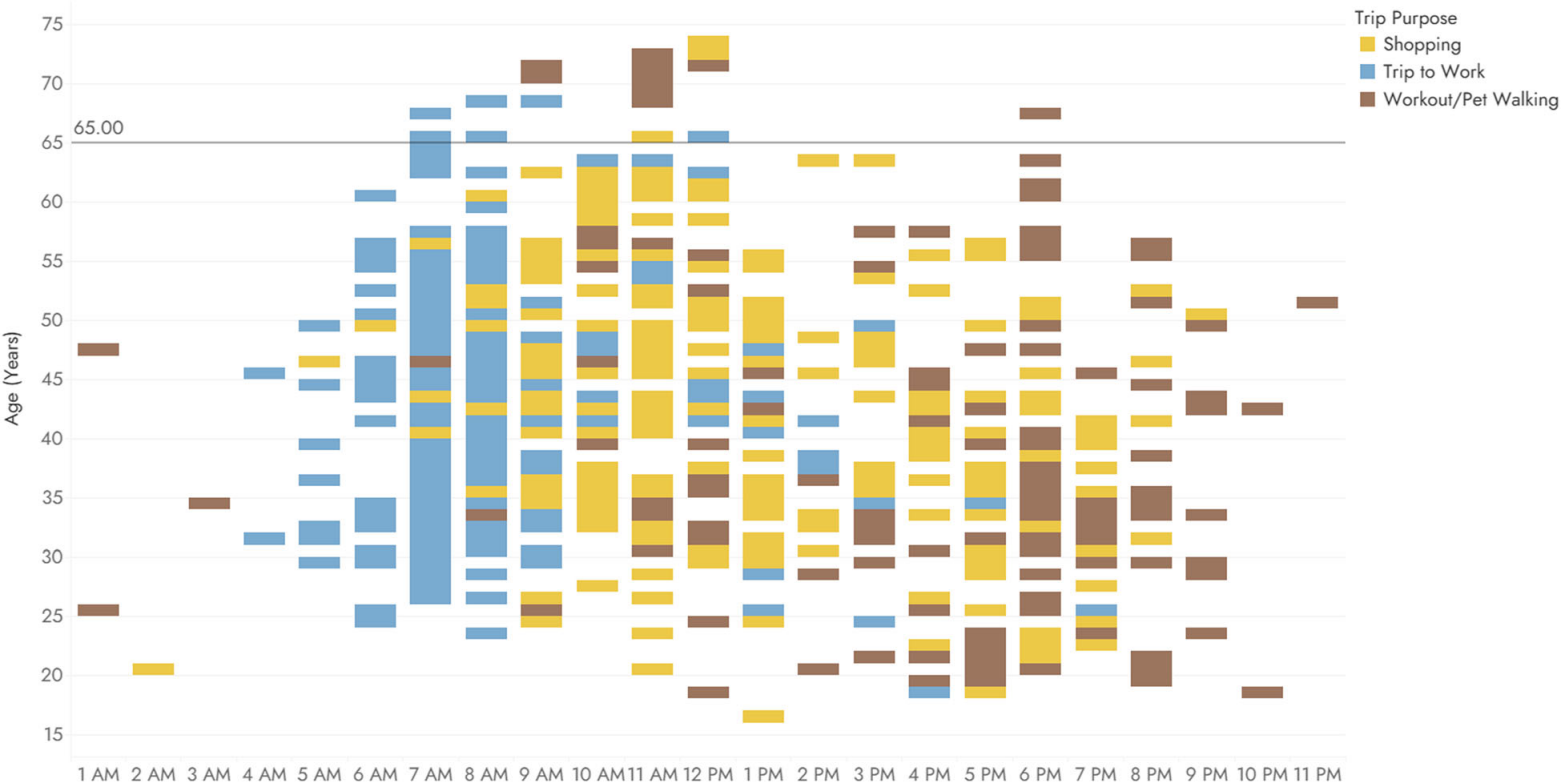
Trips throughout the day per trip purpose in relation to age

Figure 5 illustrates the trip preferences of the examined age groups throughout a day, in reference to the three basic trip purposes, shopping, commuting and workout/pet walking under the lockdown circumstances. It shows four distinct time-windows, which highlight that trips for work mainly occurred during early morning hours (5–8 am), trips for workout are mostly observed during the evening (5–9 pm), while trips for shopping purposes occur in two time-windows, in the morning (9 am – 1 pm) and in the afternoon (3–5 pm). Furthermore, users between the ages of 60 and 69 prefer to shop during the off-peak hours and especially in the first half of the day. Generally, users over the age of 65, several of whom may belong to the so-called vulnerable groups either because of age or due to various health problems, seem to focus all their activities in the morning, instead of other age groups, which spread their activities throughout the day.

#### Transport mode

In terms of the transport mode used during their last trip as stated by the respondents, it was found that the ages 30–39 moved more on foot while the ages 40–49 preferred the private car for their journeys (Kruskal – Wallis correlation test, H (6) = 23.216, *p* = 0.001) during the lockdown period.

**Fig. 6:**
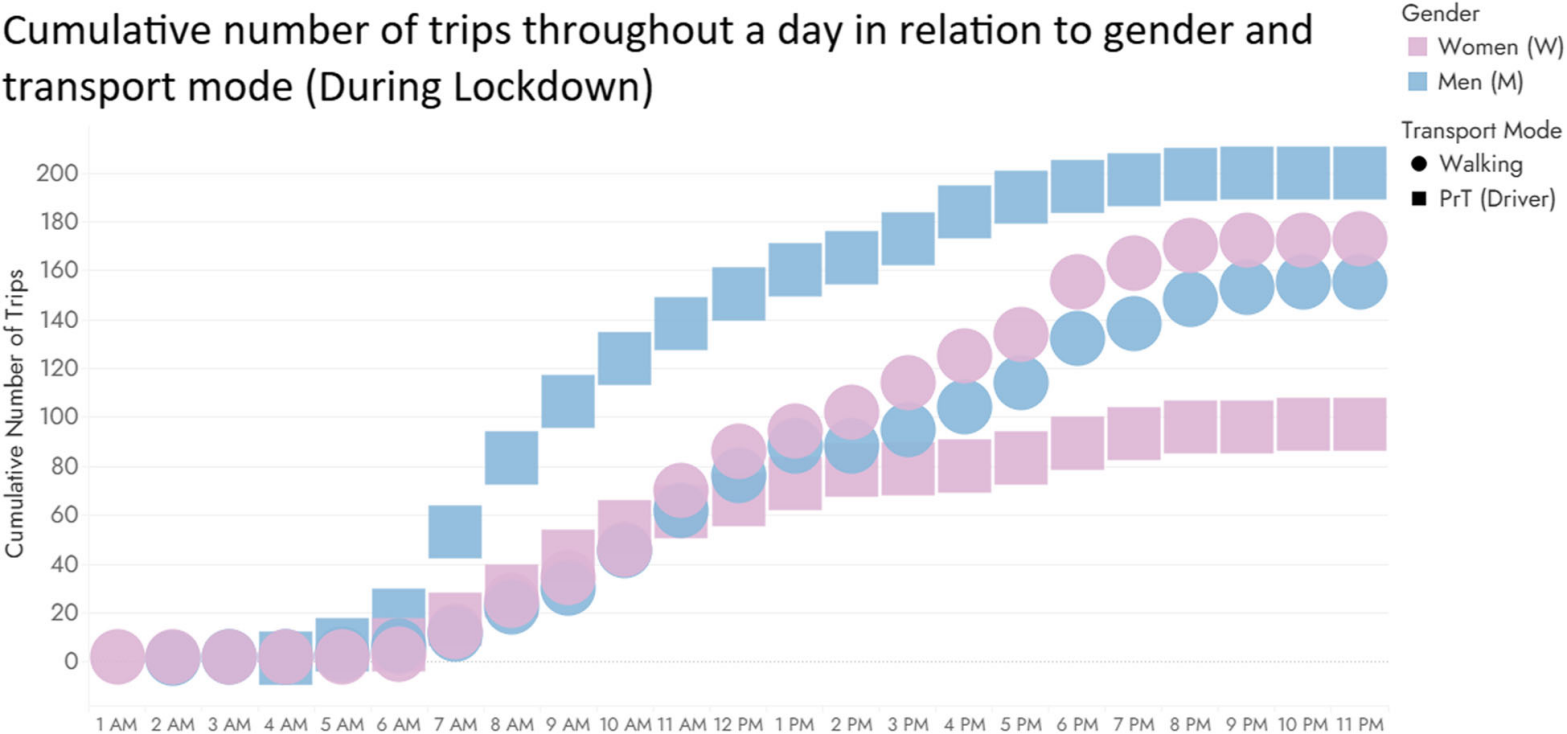
Cumulative number of trips throughout a day in relation to gender and transport mode

Survey results also indicated variations in transport mode choice based on gender. Figure 6 depicts the cumulative number of trips made during the day in relation to gender and the transport mode used (private car as driver and walking).

Our empirical findings showed that overall, during the lockdown, women preferred to travel more on foot, whereas men used their private car more (X^2^ (10, *N* = 689) = 54.218, *p* = 0.000). The daily trip profile illustrated in Fig. 6 shows that men used to complete their trips as car drivers in a wider time window (7 am – 8 pm) while the respective time window for women is narrower (8 am – 2 pm). Concerning walking there are not significant differences considering gender, as Fig. 6 indicates, regardless trip purpose.

#### Perception of safety and security

The perception of safety and security during and before the lockdown was also examined in the context of the present study (Table 6 in Appendix). Based on user responses, it appears that most respondents (57.7%), continued to shop in the same stores as before the pandemic occurred, 19.7% of them chose to visit shops nearby while only 8.3% chose shops at a greater distance due to (possibly) improved health safety conditions. More than 70% of respondents chose to do their shopping only on days and hours where there was no significant commercial traffic. Characteristic of the feeling of insecurity felt by citizens during lockdown is the fact that even when they moved outdoors their total travel time was shorter than in the normal period, at more than 52% of the responses. This may also be related to the fact that approximately 15% of the sample felt less secure when moving outdoors due to reduced traffic on the roads.

The perception of security and safety was significantly differentiated between genders. Safety-wise, women chose to shop not during peak hours (X^2^ (2, *N* = 943) = 15.275, *p* = 0.000). With regard to security, the imposed restriction of movements, resulted in a feeling of insecurity, which was especially expressed by women (X^2^ (2, *N* = 658) = 8.344, *p* = 0.015). The initial hypothesis that women appear to feel less safe and secure is confirmed by these results.

## Conclusions

The latest pandemic showed that modern communities were not well prepared to undertake the various challenges that arise, in social, economic and political perspective. This paper tries to shade light in the different mobility profiles that emerged nationwide in Greece, a country with a significantly low number of COVID-19 cases and deaths during the first way of the pandemic. The outcomes derived from the analysis are focusing primarily on the social (e.g. age, gender) and economic (e.g. income) perspectives of mobility behavior.

Outcomes of the present paper indicate an evident shift in mobility patterns of Greek citizens, in compliance to the lockdown measures imposed by the government. The number of trips completed was significantly reduced, while the choice of transport mode was also influenced by the spread of the coronavirus, with travelers choosing to avoid public transport in favour of more private means of transport, such as their car.

The implemented survey showed differentiation in mobility patterns, in relation to certain characteristics, such as gender, age or income. More to the point, men appeared to be more exposed to the virus, since they completed more trips during the lockdown period, in relation to women. Female travelers may have been more reluctant to travel, since reduced traffic, contributed to anxiety due to a low sense of safety and security. These findings indicate a possible widening of inequality across gender, as women may compromise their employment opportunities if they have to stay home to care for children. These concerns are further heightened by the fact that women tend to be employed in contact-intensive sectors, such hospitality, personal care and retail, that have been more severely impacted by the pandemic.

Furthermore, elderly travelers (over 65 years old), which are among the most vulnerable groups, adjusted their mobility needs, in order to avoid congestion in shops and services. But other than shopping during off-peak hours, elderly people seemed not to have reduced their daily trips to a large extent, as encouraged by the government and respective policy measures. Elderly people continued to travel as they have needs that cannot be fulfilled in another away, such as shopping online, video calls with friends and families, bank transactions with the use of internet, etc. The analysis also showed that lockdown led to a stronger reduction in the mobility of younger people. This could be interpreted by the fact that universities adopted e-learning as a measure early in the pandemic, as well as by the closure of bars and restaurants, thus leaving many young people that work in those sectors unemployed.

Income was proven as an influencing factor as well, since travelers that earned more, reduced their trips accordingly. People of low-income groups households are more expected to have manual labor jobs and thus continued to commute even during the pandemic period. On the contrary, people belonging to high-income groups are more likely to have an occupation that require computer skills which in turn enable them to shift to teleworking during the lockdown period.

Overall, the fact that different demographic groups reacted differently during the lockdown period calls into question the assumption that population can be treated as homogeneous. Future policies and measures should take into account this heterogeneity and act accordingly.

Results of this paper could provide policy recommendations to various stakeholders (health professionals, mobility experts, local political authorities etc.) regarding the design and planning of lockdown measures during any similar situation in the near future and contribute to the limitation of new cases and deaths. Social distancing measures, along with their respective movement restrictions, have clear direct positive effects on public health, but they are ethically challenging with human rights, because communities’ containment conflicts with individual rights of liberty and self-determination.

The findings provide food for thought, discussion and action on how the mobility sector can contribute to mitigating the effects on groups in society that are affected by the measures the most. It is clear that future policies and strategies for the mitigation of the COVID-19 pandemic effects, should take into account certain social groups, such as the elderly, in order to protect them from increased exposure to the pandemic. Furthermore, results could indicate directions for employment and financial strategies, that would target workers with low income, in order to “incentivize” them, to reduce their trips that are related to work. Targeted policy intervention is also required to support women during the pandemic, for example by offering parental leave to both men and women to encourage equal burden sharing in caring for children when schools are closed and subsequently contribute to the preservation of women’s employment opportunities.

Access - to people, goods, services e.g. shops, education, and to work (and income), healthcare, recreation – should be the key purpose of the urban transport system under the pandemic era. In the new era, accessibility and not mobility should be at the heart of economic and social welfare. Being able to reach people, goods, services is affected by transport and contemporary society’s economic and social activities are enabled and defined by urban transport systems. More specifically, addressing the psychological consequences of fear, confinement and forced cohabitation or loneliness are strongly associated with the ability of people to have access to various activities with the use of the transport system. Measures and policies after the pandemic should also give more emphasis on special user groups and especially to people with mobility needs, elderly and other vulnerable citizens such as women, homeless, mobility-impaired who often feel that they are at the margin of our today’s societies and whose needs may vary especially under exceptional circumstances.

The limitations of the present research lie mostly to the distribution of the survey sample composition. Potentially, a more detailed survey could contribute to the identification of clearer mobility patterns, but this was not possible due to the time restrictions that the relaxation of the taken measures imposed. This effort could act as groundwork for future research into the effects of socioeconomic characteristics to mobility behaviour under emergency conditions and for research into the effects of the pandemic’s second or third wave in relation to the first one.

## Data Availability

The datasets used and/or analysed during the current study are available from the corresponding author on reasonable request.

## Appendix

**Table 5:** Descriptive characteristics of main scale variables

Variable	Description	Min	Max	Mean	Standard Deviation	Variance
Age	What is your age?	16	87	41.1	12.8	163.84
Trip Start Time	At what time did your trip start?	–	–	–	–	–
Trip End Time	At what time did your trip end?	–	–	–	–	–
Total Number of Trips - Lockdown	Previous Week: Total weekly number of trips made for all trip purposes	0	22	6.51	4.48	20.09
Total Number of Trips - Typical	Typical Week: Total weekly number of trips made for all trip purposes	0	43	13.02	5.87	34.49
Trips for Commuting - Lockdown	Previous Week: Total weekly number of trips made for transition to work	0	88	1.87	3.60	12.96
Trips for Commuting - Typical	Typical Week: Total weekly number of trips made for transition to work	0	184	5.41	7.70	59.29
Trips for Commuting - Lockdown - Typical	Difference in the number of trips made for commuting before and during the lockdown measures	−170	7	−3.54	6.56	43.03
Trips Shopping - Lockdown	Previous Week: Total weekly number of trips for transition to an essential goods store	0	66	1.87	2.60	6.76
Trips Shopping - Typical	Typical Week: Total weekly number of trips for transition to an essential goods store	0	61	3.64	4.20	17.64
Trips for Shopping - Lockdown - Typical	Difference in the number of trips made for shopping before and during the lockdown measures	−60	60	−1.76	4.40	19.36
Trips Exercise - Lockdown	Previous Week: Total weekly number of trips for outdoors physical exercise or pet walking	0	25	2.21	3.10	9.61
Trips Exercise - Typical	Typical Week: Total weekly number of trips for outdoors physical exercise or pet walking	0	55	3.9	4.40	19.36
Trips for Exercise - Lockdown - Typical	Difference in the number of trips made for exercise before and during the lockdown measures	−50	15	−1.72	3.73	13.88

**Table 6:** Descriptive characteristics of main nominal/ordinal variables

Variable	Description	Range	Frequency	Type of Variable
Gender	To which gender identity do you most identify?	1: Female 2: Male 3: Other	1: 51.8% 2: 48.1% 3: 0.01%	Nominal
Income	To which income class do you categorize yourself?	1: < 1000€ 2: 1000-2000€ 3: > 2000€	1: 33.5% 2: 40.3% 3: 26.2%	Ordinal
Residence	Your current residence is located in a (large urban center, city, town etc)	1: Large urban center (population over 100,000) 2: City with a population of 50,000 to 100,000 3: City with a population of 10,000 to 50,000 4: Town with a population of 2000 to 10,000 5: Settlement with a population of less than 2000	1: 42.6% 2: 22.5% 3: 19.5% 4: 9.1% 5: 6.4%	Ordinal
Education Level	What is your education level?	1: Didn’t graduate elementary school 2: Elementary School Graduate 3: Secondary School Graduate 4: Highschool / Technical school graduate 5: University Graduate 6: Master’s Degree / PhD	1: 0.1% 2: 0.3% 3: 1.5% 4: 26.6% 5: 40.6% 6: 30.9%	Ordinal
Trip Origin	From where did you trip start?	1: From home 2: From my workplace 3: No Trip 4: Other	1: 53.9% 2: 0.3% 3: 45.3% 4: 0.5%	Nominal
Trip Purpose	What was the purpose of your trip?	1: Bank 2: Doctor/Pharmacy 3: Other 4: Provision of assistance to someone in need 5: Return home 6: Shopping 7: Trip related to work 8: Trip to work 9: Workout/Pet Walking	1: 1.0% 2: 1.4% 3: 1.7% 4: 4.3% 5: 0.2% 6: 14.0% 7: 2.5% 8: 15.6% 9: 14.0%	Nominal
Transport Mode	Which mode of transport was used for your trip?	1: Bicycle 2: Motorcycle 3: On foot 4: Private Car as a driver 5: Private Car as a passenger 6: Public Transport 7: Semi Truck 8: Special Bus 9: Taxi as a passenger 10: Truck 11: Other	1: 0.8% 2: 1.0% 3: 26.1% 4: 23.7% 5: 2.0% 6: 0.5% 7: 0.2% 8: 0.2% 9: 0.2% 10: 0.1% 11: 0.1%	Nominal
Trip Destination	Where did the trip end?	1: Bank 2: Food stores 3: Friend’s/relative’s home 4: Home 5: Outdoors location 6: Pharmacy/Doctor 7: Workplace 8: Other	1: 1.0% 2: 13.6% 3: 4.7% 4: 0.6% 5: 14.2% 6: 1.5% 7: 17.7% 8: 1.4%	Nominal
Transport Mode for Commuting - Typical	Transport mode for transition to work before lockdown movement restrictions were in effect	1: Bicycle 2: Motorcycle 3: On foot 4: Private Car as a driver 5: Private Car as a passenger 6: Public Transport 7: Special Bus 8: Taxi as a passenger	1: 0.8% 2: 2.0% 3: 8.3% 4: 24.9% 5: 2.3% 6: 3.1: 7: 0.8% 8: 0.2%	Nominal
Transport Mode for Commuting - Lockdown	Transport mode for transition to work after lockdown movement restrictions were in effect	1: Bicycle 2: Motorcycle 3: On foot 4: Private Car as a driver 5: Private Car as a passenger 6: Public Transport 7: Special Bus 8: Taxi as a passenger	1: 0.6% 2: 1.6% 3: 9.6% 4: 26.7% 5: 2.4% 6: 1.1% 7: 0.2% 8: 0.2%	Nominal
Transport Mode - Shopping - Typical	Transport more for transition to an essential goods store before lockdown movement restrictions were in effect	1: Bicycle 2: Motorcycle 3: On foot 4: Private Car as a driver 5: Private Car as a passenger 6: Public Transport 7: Special Bus 8: Taxi as a passenger	1: 1.3% 2: 2.9% 3: 31.2% 4: 44.2% 5: 4.2% 6: 1.8% 7: 0.0% 8: 0.2%	Nominal
Transport Mode - Shopping - Lockdown	Transport mode for transition to an essential goods store after lockdown movement restrictions were in effect	1: Bicycle 2: Motorcycle 3: On foot 4: Private Car as a driver 5: Private Car as a passenger 6: Public Transport 7: Special Bus 8: Taxi as a passenger	1: 1.3% 2: 1.9% 3: 35.8% 4: 40.9% 5: 5.6% 6: 0.2% 7: 0.1% 8: 0.0%	Nominal
Stores- Preferences-Lockdown	Do you prefer the same stores as the ones you did before the lockdown movements restrictions were in effect?	1: Didn’t make this kind of trip during the previous week 2: Shops in a longer distance 3: Shops in a shorter distance 4: The same shops	1: 14.2% 2: 8.3% 3: 19.7% 4: 57.7%	Nominal
Perception of Safety	Do you prefer to visit stores during days and hours that you believe the store will be less crowded?	1: Didn’t make this kind of trip during the previous week 2: Shop only during low traffic days and hours 3: Shop during any day or hour	1: 14.2% 2: 74.9% 3: 10.9%	Nominal
Perception of Security	Do you feel secure outdoors due to the streets being less crowded, compared to before the lockdown movement restrictions were in effect?	1: I feel as secure as before 2: I feel less secure 3: I feel more secure	1: 52.3% 2: 14.7% 3: 33.0%	Nominal
